# Organo-fluorine chemistry III

**DOI:** 10.3762/bjoc.9.255

**Published:** 2013-10-23

**Authors:** David O'Hagan

**Affiliations:** 1 School of Chemistry, University of St Andrews, Fife, KY16 9ST, United Kingdom

**Keywords:** organo-fluorine

It is a pleasure to introduce the 3^rd^ in the series of Thematic Issues on organo-fluorine chemistry for the Beilstein Journal of Organic Chemistry (BJOC). These series have become progressively more successful over time in terms of the number and quality of the contributions received. It is also pleasing to note that the profile and impact factor of the journal has increased steadily over this time as well.

Organo-fluorine chemistry is enjoying a high profile at present, particularly in methodology development. There has been considerable success across the international community in the development of organometallic methods for the incorporation of fluorinated substituents onto aryl and heterocyclic rings. Such motifs are required by the pharmaceutical and agrochemical industries to modulate the performance and pharmacokinetics of bioactives. The particular challenge of delivering direct and efficient methods for C–F, C–R_f_ or C–XR_f_ bond formation has been a major focus in the last few years and landmark developments have been made. Many of the contributions in the Thematic Series focus on this aspect of organo-fluorine chemistry.

Fluorine touches all categories of performance compounds extending from bioactives to organic materials, and society demands continual improvements in the quality and performance of products and devices. As the global population steadily increases the pressure to improve health care and agricultural yields, is immense. This requires innovation in molecular design, and because of its particular ability to tune properties, fluorine chemistry may provide the means. To meet these demands we continually need new methods and new building blocks to prepare new classes of compounds. As our ability to prepare and apply organo-fluorine molecules improves, it demands a deeper understanding of their properties and behaviour. The papers of the Thematic Series touch on all of these aspects and the area remains as innovative and relevant as ever.

I have to thank all of the authors who have taken the time to prepare manuscripts, and to offer their latest scientific contributions to this Thematic Series. The quality of the papers and the geographical distribution of the contributing laboratories is a clear indication of the health, vitality and importance of fluorine chemistry as a specialism within the wider chemical enterprise.

David O'Hagan

St Andrews, September 2013

